# Mutation Processes in 293-Based Clones Overexpressing the DNA Cytosine Deaminase APOBEC3B

**DOI:** 10.1371/journal.pone.0155391

**Published:** 2016-05-10

**Authors:** Monica K. Akre, Gabriel J. Starrett, Jelmar S. Quist, Nuri A. Temiz, Michael A. Carpenter, Andrew N. J. Tutt, Anita Grigoriadis, Reuben S. Harris

**Affiliations:** 1 Department of Biochemistry, Molecular Biology, and Biophysics, Institute for Molecular Virology, Masonic Cancer Center, University of Minnesota, Minneapolis, MN, United States of America; 2 Breast Cancer Now Research Unit, Research Oncology, Guy’s Hospital, King’s College London, London, United Kingdom; 3 Howard Hughes Medical Institute, University of Minnesota, Minneapolis, MN, United States of America; Institut de Recherches Cliniques de Montréal (IRCM), CANADA

## Abstract

Molecular, cellular, and clinical studies have combined to demonstrate a contribution from the DNA cytosine deaminase APOBEC3B (A3B) to the overall mutation load in breast, head/neck, lung, bladder, cervical, ovarian, and other cancer types. However, the complete landscape of mutations attributable to this enzyme has yet to be determined in a controlled human cell system. We report a conditional and isogenic system for A3B induction, genomic DNA deamination, and mutagenesis. Human 293-derived cells were engineered to express doxycycline-inducible A3B-eGFP or eGFP constructs. Cells were subjected to 10 rounds of A3B-eGFP exposure that each caused 80–90% cell death. Control pools were subjected to parallel rounds of non-toxic eGFP exposure, and dilutions were done each round to mimic A3B-eGFP induced population fluctuations. Targeted sequencing of portions of *TP53* and *MYC* demonstrated greater mutation accumulation in the A3B-eGFP exposed pools. Clones were generated and microarray analyses were used to identify those with the greatest number of SNP alterations for whole genome sequencing. A3B-eGFP exposed clones showed global increases in C-to-T transition mutations, enrichments for cytosine mutations within A3B-preferred trinucleotide motifs, and more copy number aberrations. Surprisingly, both control and A3B-eGFP clones also elicited strong mutator phenotypes characteristic of defective mismatch repair. Despite this additional mutational process, the 293-based system characterized here still yielded a genome-wide view of A3B-catalyzed mutagenesis in human cells and a system for additional studies on the compounded effects of simultaneous mutation mechanisms in cancer cells.

## Introduction

Cancer genome sequencing studies have defined approximately 30 distinct mutation signatures (reviewed by [1–4]). Some signatures are large-scale confirmations of established sources of DNA damage that escaped repair or were repaired incorrectly. The largest is water-mediated deamination of methyl-cytosine bases, which manifest as C-to-T transitions in genomic 5’-CG motifs [5]. This process impacts almost all cancer types and accumulates as a function of age. Other well known examples include ultraviolet radiation, UV-A and UV-B, which crosslink adjacent pyrimidine bases and result in signature C-to-T transitions [6], and tobacco mutagens such as nitrosamine ketone (NNK), which metabolize into reactive forms that covalently bind guanine bases and result in signature G-to-T transversions [7]. These latter mutagenic processes are well known drivers of skin cancer and lung cancer, respectively, but also contribute to other tumor types. A lesser-known but still significant example of a mutagen is the dietary supplement aristolochic acid, which is derived from wild ginger and related plants and metabolized into reactive species that covalently bind adenine bases and cause A-to-T transversions [8, 9]. Aristolochic acid mutation signatures are evident in urothelial cell, hepatocellular, and bladder carcinomas. Other confirmed mutation sources include genetic defects in recombination repair (*BRCA1*, *BRCA2*, etc.), post-replication mismatch repair (*MSH2*, *MLH1*, etc.), and DNA replication proofreading function, which manifest as microhomology-mediated insertion/deletion mutations, repeat/microsatellite slippage mutations, and transversion mutation signatures, respectively [4, 5, 10].

The largest previously undefined mutation signature in cancer is C-to-T transitions and C-to-G transversions within 5’-TC dinucleotide motifs [5, 11, 12]. This mutation signature occurs throughout the genome, as well as less frequently in dense clusters called kataegis. This signature is ascribable to the enzymatic activity of members of the APOBEC family of DNA cytosine to uracil deaminases [5, 11–15]. Human cells encode up to 9 distinct APOBEC family members with demonstrated C-to-U editing activity, and 7/9 have been shown to prefer 5’-TC dinucleotide motifs in single-stranded DNA substrates: APOBEC1, APOBEC3A, APOBEC3B (A3B), APOBEC3C, APOBEC3D, APOBEC3F, and APOBEC3H. In contrast, AID and APOBEC3G prefer 5’RC and 5’CC, respectively (R = purine; reviewed by [16, 17]). The size and similarity of this protein family, as well as the formal possibility that another DNA damage source may be responsible for the same mutation signature [18], have made DNA sequencing data and informatics analyses open to multiple interpretations.

However, independent [13, 19] and subsequent [14, 15, 20–26] studies indicate that at least one DNA deaminase family member, A3B, has a significant role in causing these types of mutations in cancer. A3B localizes to the nucleus throughout the cell cycle except during mitosis when it appears excluded from chromatin [19]. A3B is upregulated in breast cancer cell lines and primary tumors at the mRNA, protein, and activity levels [13, 20, 27]. Endogenous A3B is the only detectable deaminase activity in nuclear extracts of many cancer cell lines representing a broad spectrum of cancer types (breast, head/neck, lung, ovarian, cervix, and bladder [13, 20, 27]). Endogenous A3B is required for elevated levels of steady state uracil and mutation frequencies in breast cancer cell lines [13]. Overexpressed A3B induces a potent DNA damage response characterized by gamma-H2AX and 53BP1 accumulation, multinuclear cell formation, and cell cycle deregulation [13, 21, 22]. A3B levels correlate with overall mutation loads in breast and head/neck tumors [13, 23]. The biochemical deamination preference of recombinant A3B, 5’TCR, is similar to the actual cytosine mutation pattern observed in breast, head/neck, lung, cervical, and bladder cancers [13, 14, 20]. Human papillomavirus (HPV) infection induces A3B expression in several human cell types, providing a link between viral infection and the observed strong APOBEC mutation signatures in cervical and some head/neck and bladder cancers [28–30]. The spectrum of oncogenic mutations in *PIK3CA* is biased toward signature A3B mutation targets in HPV-positive head/neck cancers [23]. Last but not least, high *A3B* levels correlate with poor outcomes for estrogen receptor-positive breast cancer patients [25, 26, 31].

Despite this extensive and rapidly growing volume of genomic, molecular, and clinical information on A3B in cancer, the association between A3B and APOBEC mutational signatures has so far only been correlative, and a mechanistic demonstration of this enzyme’s activity on the human genome has yet to be determined. Here we report further development of a human 293 cell-based system for conditional expression of human A3B. The results reveal, for the first time in a human cell line, the genomic landscape of A3B induced mutagenesis.

## Materials and Methods

### Cell Lines

We previously reported T-REx-293 cells that conditionally express A3B [13]. However, the mother, daughter, and granddaughter lines described here are new in order to ensure a single cell origin and have all of the controls derived in parallel. T-REx-293 cells were cultured in high glucose DMEM (Hyclone) supplemented with 10% FBS and 0.5% Pen/Strep. Single cell derived mother lines, A and C, were obtained by limiting dilution in normal growth medium. These mother clones were transfected with linearized pcDNA5/TO-A3Bintron-eGFP (A3Bi-eGFP) or pcDNA5/TO-eGFP vectors [13, 32], selected with 200 μg/mL hygromycin, and screened as described in the main text to identify drug-resistant daughter clones capable of Dox-mediated induction of A3Bi-eGFP or eGFP, respectively. The encoded A3B enzyme is identical to “isoform a” in GenBank (NP_004891.4). GFP flow cytometry was done using a FACSCanto II instrument (BD Biosciences).

### Immunoblots

Whole cell lysates were prepared by suspending 1x10^6^ cells in 300μL 10x reducing sample buffer (125mM Tris pH 6.8, 40% Glycerol, 4%SDS, 5% 2-mercaptoethanol and 0.05% bromophenol blue). Soluble proteins were fractionated by 4% stacking and 12% resolving SDS PAGE, and transferred to PVDF membranes using a wet transfer BioRad apparatus. Membranes were blocked for 1 hr in 4% milk in PBS with 0.05% sodium azide. Primary antibody incubations, anti-GFP (JL8-BD Clontech) and anti-β-actin (Cell Signaling) were done in at a 1:1000 dilution in 4% milk diluted in PBST, and incubation conditions ranged from 4–8 degrees C for 2–16 hrs. Membranes were then washed 3 times for 5 minutes in PBST. Secondary antibody incubations, anti-mouse 680 (1:20000) and anti-rabbit 800 (1:20000), were done in 4% milk diluted in PBST with 0.01% SDS, and incubation conditions ranged from 4–8 degrees C for 2–16 hrs. The resulting membranes were washed 3 times for 5 minutes in PBST and imaged using Licor instrumentation (Odyssey).

### DNA Deaminase Activity Assays

This assay was adapted from published procedures [27, 33]. Whole-cell extracts were prepared from 1x10^6^ cells by sonication in 200μL HED buffer (25mM HEPES, 5mM EDTA, 10% glycerol, 1mM DTT, and one tablet protease inhibitor-Roche per 50mL HED buffer). Debris was removed by a 30 min maximum speed spin in a tabletop micro-centrifuge at 4 degrees C. The supernatant was then used in 20μL deamination reactions that contained the following: 1μL of 4pM fluorescently-labeled 43-mer oligo (5’-ATTATTATTATTCGAATGGATTTATTTATTTATTTATTTATTT-fluorescein) containing a single interior 5’-TC substrate, 9.25μL UDG (NEB), 0.25μL RNase, 2μL 10x UDG buffer (NEB), 16.5μL lysate. Reactions were incubated at 37 degrees C for 1h. 2μL 1M NaOH was added and reaction was heated to 95 degrees C in a thermocycler for 10 min. 22μL of 2x formamide loading buffer was added to each sample. 5μL of each reaction was fractionated on a 15% TBE Urea Gel and imaged using a SynergyMx plate reader (BioTek).

### Differential DNA Denaturation (3D) PCR Experiments

This assay was adapted from published procedures [13, 34]. Genomic DNA was extracted from samples using a PureGene protocol (Gentra) and quantified using Nanodrop instrumentation (ThermoFisher Scientific). 20 ng of genomic DNA was subjected to one round of normal high denaturation temperature PCR using Taq Polymerase (Denville) and primers for *MYC* (5’-ACGTTAGCTTCACCAACAGG and 3’TTCATCAAAAACATCATCATCCAG) or *TP53* (5’GAGCTGGAGCTTAGGCTCCAGAAAGGACAA and 3’TTCCTAGCACTGCCCAACAACACCAGC). 383 bp and 376 bp PCR products were purified and quantified using qPCR with nested primer sets and SYBR Green detection (Roche 480 LightCycler; 5’ACGAGGAGGAGAACTTCTACCAGCA and 3’TTCATCTGCGACCCGGACGACGAGA for *MYC* and 5’TTCTCTTTTCCTATCCTGAGTAGTGGTAA and 3’TTATGCCTCAGATTCACTTTTATCACCTTT for *TP53*). Equivalent amounts of each PCR product were then used for 3D-PCR using the same nested PCR primer sets. The resulting 291 and 235 bp products were fractionated by agarose gel electrophoresis, purified using QIAEX II (Qiagen), cloned into a pJet vector (Fermentas), and subjected to sequencing (GENEWIZ). Alignments and mutation calls were done with Sequencher (Gene Codes Corporation).

### SNP Array Based Mutational Analysis

Granddaughter clones were established by limiting dilution after the final pulse round. Genomic DNA was prepared from daughter and granddaughter clones using the Gentra PureGene kit (Qiagen, Valencia, CA), quantified by agarose gel staining with ethidium bromide and by NanoDrop measurements (Thermo Scientific, Wilmington, DE), and subjected to SNP array analyses by Source BioScience (Cambridge, UK) using the Human OmniExpress-24v1-0 BeadChip (Illumina, San Diego, CA). Raw data were pre-processed in GenomeStudio using the Genotyping Module (Illumina, San Siego, CA). Genotype clustering was performed using the *humanomniexpress_24v1-0_a* cluster file, whereby probes with a GenCall score below 0.15, indicating low genotyping reliability, were discarded. All samples passed quality control as assessed by call rates and frequencies. Genotypes for a total of 716,503 probes were used for further analyses.

By comparing the genotypes of the granddaughter clones to the pre-pulsed daughter clones, six classes of base substitutions could be determined (C-to-T, C-to-G, C-to-A, T-to-G, T-to-C, and T-to-A). For example, a C-to-T transition occurred if the C/C genotype of the mother clone changed to a C/T genotype in the granddaughter clone. Given the design of some microarray probes (i.e., some probes detect the Watson-strand rather than the Crick-strand), a change from a G/G in the mother clone to a G/A genotype in the granddaughter clone was also scored as a C-to-T transition.

Chromosomal abnormalities in the genomes of granddaughter clones were identified with Nexus Copy Number 7.5 software (BioDiscovery, Hawthorne, CA), using the matched mother clone as a reference. SNPRank segmentation was applied and the segmented copy number data were further processed with the Tumor Aberrations Prediction Suite (TAPS) to obtain allele-specific copy number profiles [35]. All analyses were performed using the R statistical environment (http://www.R-project.org). The number of copy number alterations in the A3B-eGFP pulsed clones were determined based on the difference between the segment copy number counts of the A3B-eGFP pulsed clones and the eGFP pulsed clones. Segments which the eGFP pulsed granddaughter clones were not identical or had CN of 0 were excluded. These were subsequently binned by copy number loss or gain. All SNP data sets have been deposited in the NCBI GEO database under accession code GSE78710.

### Whole Genome Sequencing (WGS)

Library preparation and sequencing was performed by the Beijing Genome Institute (BGI) on the Illumina X Ten platform to an average of 34.5 ± 2.8 fold coverage using purified DNA from Pulse 10 subclone extractions described in the SNP array based methods. Sequences were aligned to the hg19 reference genome using BWA. PCR duplicates were marked and removed with Picard-tools (Broad). Somatic mutation calling was conducted using mpileup (SamTools), VarScan2 (Washington University, MO)), and MuTect (Broad Institute, MA). Mutations detected by both VarScan2 and MuTect were kept as true somatic mutations. VarScan2 was run using procedures describe by de Bruin and coworkers [24]. MuTect was run using default parameters. Alignments from CG1 and CG2 were used as “normal” controls for CA1 and CA3, respectively. Alignment from AG3 was used as the as “normal” control for AA3. CG1 and CG2 were used as normals for each other in order to determine their somatic mutations. Somatic mutations that were called against multiple “normal” genomes were merged to increase detection rates by overcoming regions of poor sequence coverage unique to either “normal” genome. Variants occurring at an allele frequency greater than 0.5 or falling into repetitive regions or those with consistent mapping errors were removed as described [24]. Somatic indels were called by VarScan2 and filtered using the same methods described above. Separation of mutation signatures present in our WGS data was performed by the Somatic Signatures R package using nsNMF decomposition instead of Brunet NMF decomposition as described by Covington and colleagues [36]. Mutation strand asymmetries were analyzed using somatic mutations from all samples and the AsymTools MatLab software [37]. All raw sequences are available from NCBI SRA under project number, PRJNA312357.

## Results

### System for Conditional A3B Expression

**Fig 1:**
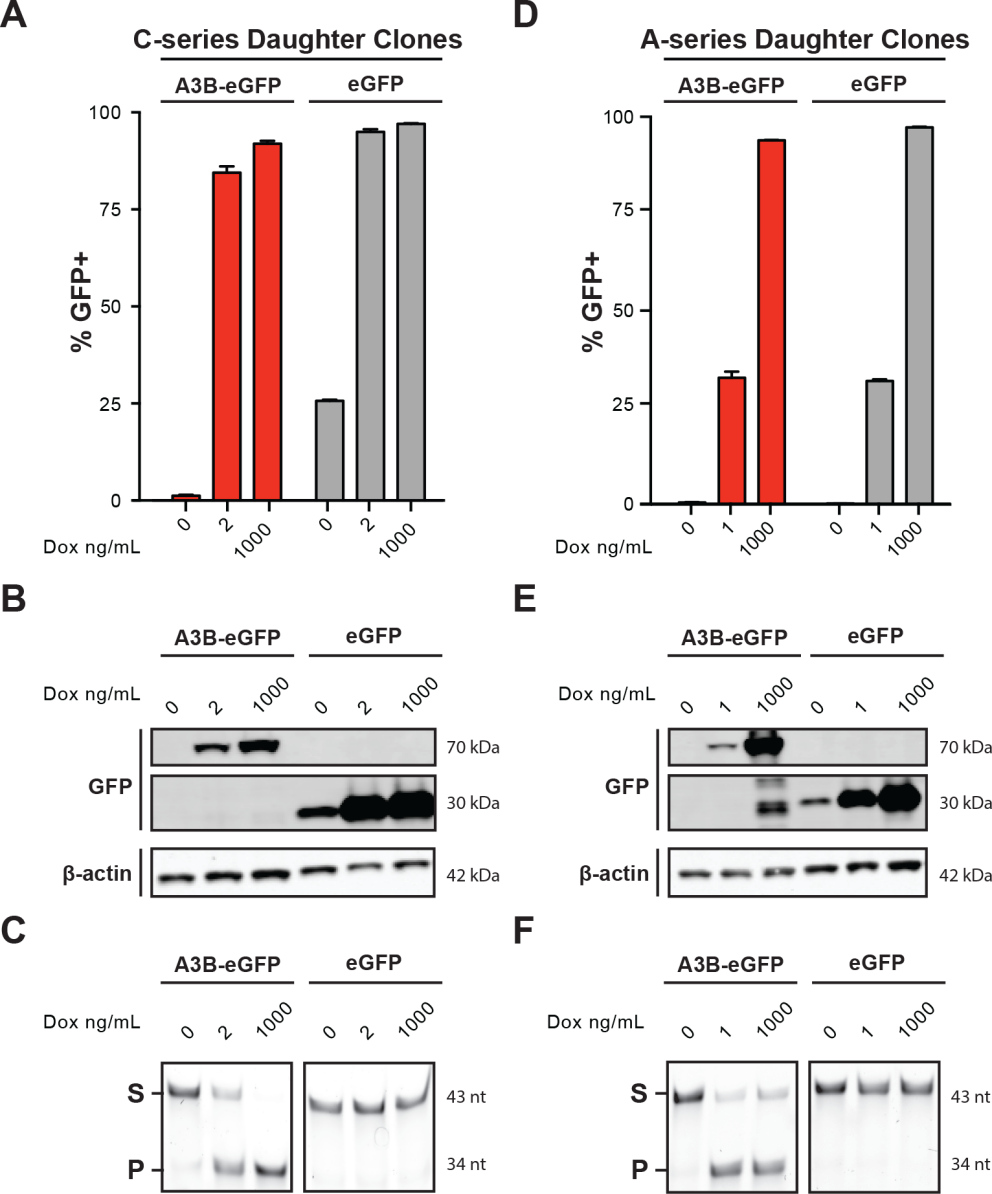
A conditional system for A3B expression. (A) Flow cytometry data for T-REx-293 A3B-eGFP and eGFP daughter cultures 24 hrs after Dox treatment (n = 3; mean +/- SD of technical replicates). (B) Anti-GFP immunoblot of T-REx-293 A3B-eGFP and eGFP daughter cultures 24 hrs after Dox treatment. (C) DNA cytosine deaminase activity data of whole cell extracts from T-REx-293 A3B-eGFP and eGFP daughter cultures 24 hrs after Dox treatment. (D, E, F) Biological replicate data using A-series daughter clones of the experiments described in panels A, B, and C, which used C-series daughter clones.

Previous studies have demonstrated that A3B over-expression induces a strong DNA damage response resulting in cell cycle aberrations and eventual cell death [13, 19, 21, 22, 32]. To be able to control the degree of A3B-induced genotoxicity, we built upon our prior studies [13] by establishing a single cell-derived isogenic system for conditional and titratable expression of this enzyme. T-REx-293 cells were subcloned to establish an isogenic “mother” line, which was then transfected stably with a doxycycline (Dox) inducible A3B-eGFP construct or with an eGFP vector as a negative control. The resulting “daughter” clones were screened by flow cytometry to identify those that were non-fluorescent without Dox (i.e., non-leaky) and uniformly fluorescent with Dox treatment (**Fig 1A**). Daughter clones were also screened for Dox-inducible overexpression of A3B-eGFP or eGFP by anti-GFP immunoblotting (**Fig 1B**). A3B-eGFP clones were uniformly GFP-negative without Dox treatment, but eGFP only clones showed a low level of leaky expression possibly related to greater protein stability. As additional confirmation, the functionality of the induced A3B-eGFP protein was tested using an *in vitro* ssDNA deamination assay using whole cell extracts [33]. As expected, only extracts from Dox-treated A3B-eGFP cells elicited strong ssDNA C-to-U editing activity as evidenced by the accumulation of the deaminated and hydrolytically cleaved reaction products (labeled P in **Fig 1C**; see Methods for details). Nearly identical results were obtained with a parallel set of independently derived daughter clones (**Fig 1D and 1E**).

### Iterative Rounds of A3B Exposure

**Fig 2:**
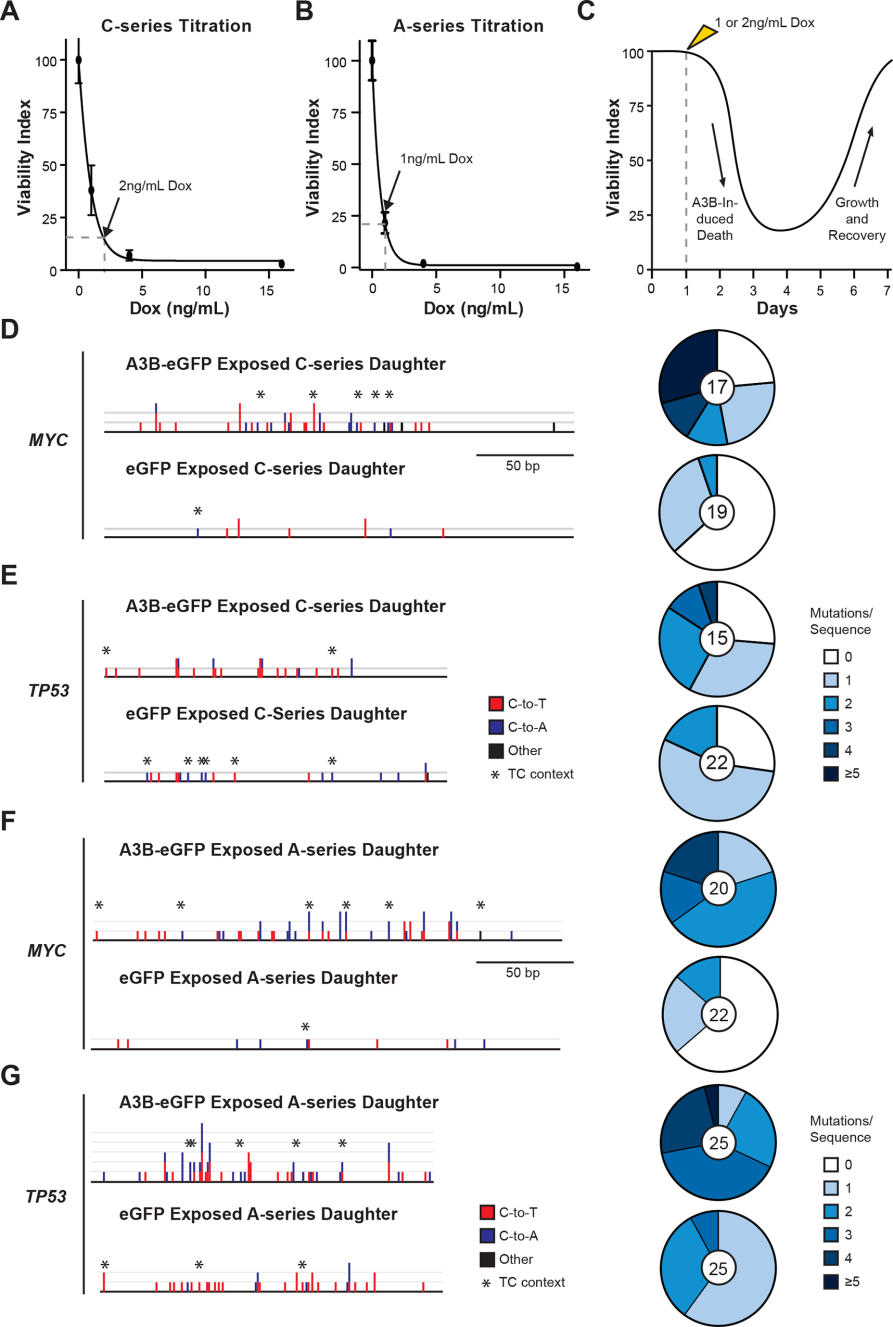
A3B induction optimization and targeted sequencing results. (A, B) Dose response curves indicating the relative colony forming efficiency (viability index) of T-REx-293 A3B-eGFP daughter clones treated with the indicated Dox concentrations (n = 3; mean viability +/- SD of biological replicates). The dotted lines show the Dox concentration required to induce 80% cell death (2 or 1 ng/mL for C- and A-series daughter clones, respectively). (C) A schematic representation of the experimental workflow depicting the viability index of a population of cells induced to express A3B-eGFP and recover over time. Dox treatment occurs on day 1, maximal death is observed on days 3 or 4, and each population typically rebounds to normal viability levels by days 6 or 7. (D-G) A summary of the base substitution mutations observed in *MYC* (241 bp) and *TP53* (176 bp) by 3D-PCR analysis of genomic DNA after 10 rounds of A3B-eGFP or eGFP exposure. Red, blue, and black columns represent the absolute numbers of C-to-T, C-to-A, and other base substitution types in sequenced 3D-PCR products, respectively. Asterisks indicate cytosine mutations occurring in 5’-TC dinucleotide motifs. The adjacent pie graphs summarize the base substitution mutation load for each 3D-PCR amplicon. The number of sequences analyzed is indicated in the center of each pie graph.

To establish reproducible A3B induction conditions, a series of cytotoxicity experiments was done using a range of Dox concentrations. 10,000 T-REx-293 A3B-eGFP cells were plated in 10 cm plates in triplicate, treated with 0, 1, 4, or 16 ng/mL Dox, incubated 14 days to allow time for colony formation, and quantified by crystal violet staining. As expected, higher Dox concentrations led to greater levels of toxicity (**Fig 2A** and 2**B**). Interpolation from a best-fit logarithmic curve indicated that 2 ng/mL Dox (C-series daughter clone) or 1 ng/mL Dox (A-series daughter clone) would cause 80–90% cytotoxicity, and this concentration was selected for subsequent experiments. Taken together with the measured doubling times of daughter clones, each A3B-eGFP induction series was estimated to span 7 days (represented in the workflow schematic in **Fig 2C**).

Each T-REx-293 A3B-eGFP daughter clone was then subjected to 10 rounds of A3B-eGFP induction and recovery (**Fig 2C**). Iterative exposures to A3B-eGFP were expected to generate dispersed mutations throughout the genome. Ten rounds of A3B-eGFP induction were chosen as a sufficient regimen for the cells to accumulate readily detectable levels of somatic mutation as a proof-of-concept for this inducible system. This approach also left open the option to go back and characterize an intermediate round, or pursue additional rounds should analyses require less or more mutations, respectively.

A potential pitfall of this experimental approach is the possibility of selecting cells that have inactivated the A3B expression construct or the capacity for induction to avoid the cytotoxic effects of overexpressing this DNA deaminase. Aliquots of cells from each pulse series were therefore periodically tested by flow cytometry for A3B-eGFP inducibility, western blot for protein expression, and ssDNA deamination assays for enzymatic activity (e.g., **Fig 1**). Even after the tenth induction series, the A3B-eGFP daughter clones performed similar to original daughter cultures as well as to daughter cultures that had been grown continuously in parallel to the Dox-exposed experimental cultures and diluted to mimic the population dynamics caused by each A3B-eGFP exposure (e.g., **Fig 1**). These observations indicate that, despite negative selection pressure imposed by A3B-eGFP mediated DNA damage, resistance or escape mechanisms did not become overt.

### Targeted DNA Sequencing Provides Evidence for A3B Mutagenesis

Next, target gene 3D-PCR and sequencing were used to determine if the cells within each daughter culture had accumulated detectable levels of mutation after 10 rounds of A3B-eGFP exposure. 3D-PCR is a technique that enables the preferential recovery of DNA templates with C-to-T transitions and/or C-to-A transversions, because these mutations cause reduced hydrogen bonding potential and yield DNA molecules that can be amplified at PCR denaturation temperatures lower than those required to amplify the original non-mutated sequences [13, 38, 39]. *MYC* and *TP53* were selected as target genes for this analysis because our prior work with transiently over-expressed A3B and by others with related A3 family members has demonstrated that these genomic regions are susceptible to enzyme-catalyzed deamination [13, 34, 40–46].

The 3D-PCR and DNA sequencing analyses revealed substantially more mutations in *MYC* and *TP53* in A3B-eGFP exposed daughter cultures in comparison to controls (**Fig 2D–2G**). For instance, in the C-series daughter clone 43 mutations, mostly C-to-T transitions, were evident in *MYC* amplicons from A3B-eGFP exposed cultures, whereas only 9 mutations were found in a similar number of control amplicons (mutation plot on left side of **Fig 2D**; p = 0.00036, Student's two-tailed t-test). The mutation load per amplicon was also higher (pie graphs on right side of **Fig 2D**). Similar results were obtained for *TP53* (**Fig 2E**; p = 0.11, Student's two-tailed t-test), as well as for both *MYC* and *TP53* in a parallel set of independently derived A-series daughter clones (**Fig 2F and 2G**; p<0.0001 and p<0.0001, respectively, Student's two-tailed t-test). The differences between A3B-eGFP exposed and control conditions were statistically significant for three of four conditions and, taken together, these results provided strong confirmation that 10 rounds of A3B-eGFP exposure caused increased levels of genomic DNA mutagenesis.

### Genome Wide Mutation Analyses

The experiments described used pools of cells and, due to the largely stochastic nature of the A3B mutational process and the duration of the pulse series, each pool would be expected to manifest extreme genetic heterogeneity. This complexity would constrain a standard deep sequencing approach by enabling only the earliest arising mutations to be detected in the pool because most subsequent mutations would persist at frequencies too low for reliable detection. To reduce this complexity to a manageable level and be able to investigate the mutational history of a single cell exposed to iterative rounds of either A3B-eGFP or eGFP, we used limiting dilution to generate “granddaughter” subclones from the tenth generation daughter pools. The strength of this strategy is that any new base substitution in a single daughter cell, which occurred between the time the daughter clone was originally generated until the recovery period following the tenth Dox treatment, would be fixed in the granddaughter clonal population at a predictable allele frequency depending on local chromosome ploidy (i.e., new mutations would be expected at 50% in diploid regions, 33% in triploid regions, 25% in tetraploid regions, etc., of the 293 cell genome).

**Fig 3:**
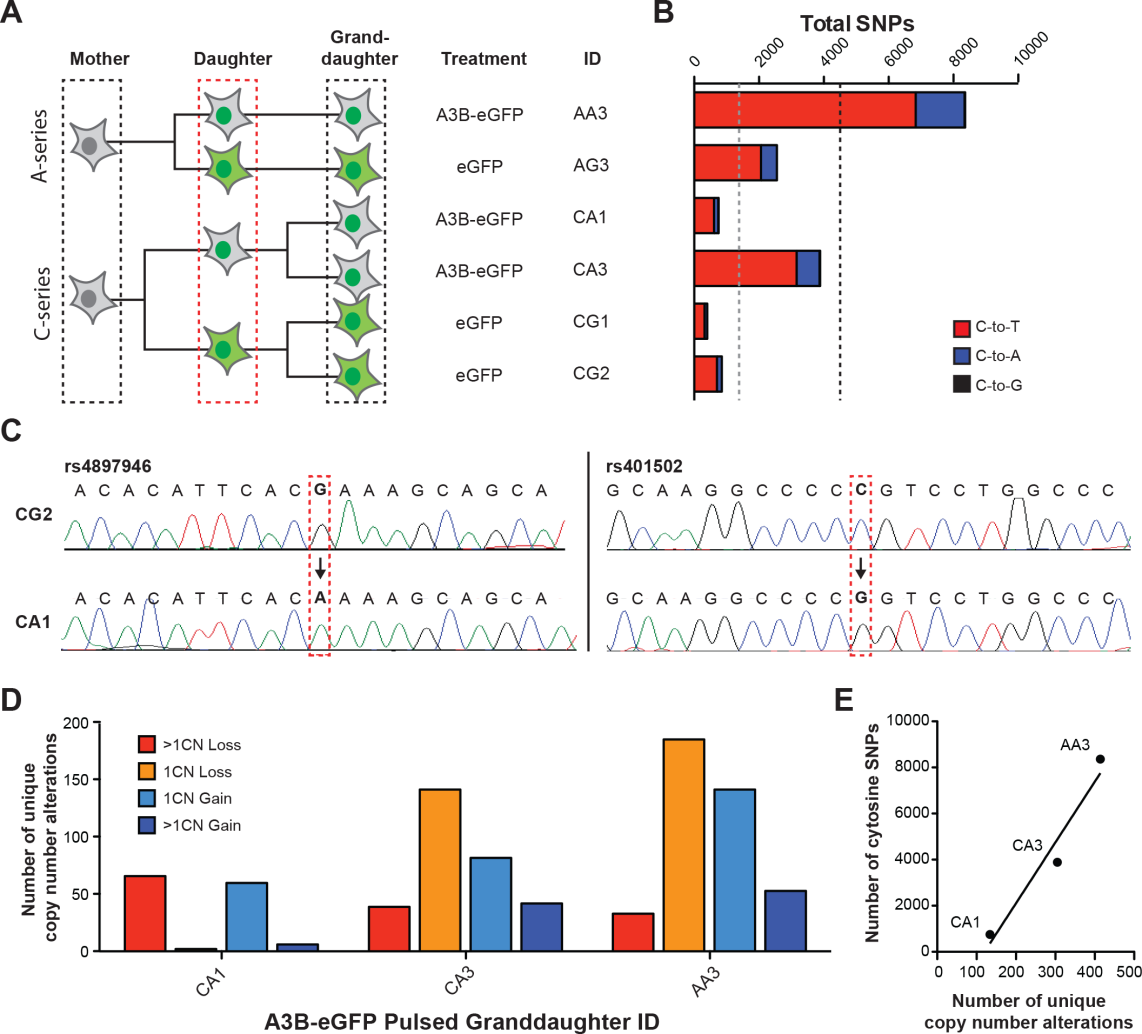
SNP analyses to estimate new mutation accumulation. (A) A dynastic tree illustrating the relationship between mother, daughter, and granddaughter clones used for SNP and WGS experiments. The red, dashed box around the daughter clones denotes 10 cycles of Dox-treatment. (B) A histogram summarizing the SNP alterations observed in granddaughter clones by microarray hybridization. Red, blue, and black colors represent C-to-T, C-to-A, and C-to-G mutations, respectively. (C) Sanger sequencing chromatograms confirming representative cytosine mutations predicted by SNP analysis. The left chromatogram shows a G-to-A transition (C-to-T on the opposite strand) and the right chromatogram a C-to-G transversion. (D) A histogram plot of the total number of copy number (CN) alterations in the indicated categories in A3B-eGFP exposed granddaughter clones in comparison to eGFP exposed controls, which were normalized to zero in order to make this comparison. (E) A dot plot and best-fit line of data in panel B versus data in panel D.

The dynastic relationship between mother, daughter, and granddaughter clones in this study is shown in **Fig 3A**. To provide initial estimates of the overall level of new base substitution mutations, genomic DNA was extracted from each granddaughter clone and subjected to single nucleotide polymorphism (SNP) analysis using the Illumina OmniExpress Bead Chip. A base substitution mutation was defined as a clear SNP difference between each daughter clone and her respective granddaughter clone. These analyses revealed a wide range of SNP alterations among granddaughter clones, ranging from a low of <500 in the C-series eGFP expressing granddaughter subclone CG1 to a high of over 8,000 in the A-series A3B-eGFP expressing granddaughter subclone AA3 (**Fig 3B**). This extensive variability was expected based on the sublethal Dox concentration used in each exposure round, the randomness of granddaughter clone selection, and the stochastic nature of the mutation processes. Nevertheless, A3B-eGFP exposed granddaughter clones had an average of 3.4-fold more new cytosine mutations than the eGFP controls (averages shown by dashed vertical lines in **Fig 3B**). Sanger sequencing of cloned PCR products was used to confirm several distinct SNP alterations and provided an orthologous validation of this array-based approach (e.g., representative chromatograms of mutations in granddaughter CA1 versus corresponding non-mutated sequences from CG2 in **Fig 3C**). In addition, hundreds more genomic copy number alterations were evident in A3B-eGFP exposed granddaughters in comparison eGFP controls (**Fig 3D**). Interestingly, the overall number of copy number alterations appeared to correlate positively with the overall number of cytosine mutations, suggesting that many A3B-catalyzed genomic DNA deamination events are likely processed into DNA breaks and result in larger-scale copy number aberrations (**Fig 3E**).

### A3B Mutational Landscape by Whole Genome Sequencing

Next, whole genome sequencing (WGS) was done to assess the mutation landscape for 3 A3B-eGFP exposed and 3 eGFP control granddaughter clones from two distinct biological replica experiments (granddaughters depicted in **Fig 3A**). Samples were sequenced using the Illumina X Ten platform at the Beijing Genome Institute. Approximately 700 million 150 bp paired-end reads were generated for each genome, with an average read depth of 34.5 ± 2.8 (SD) per locus. Reads were aligned against the hg19 genome with BWA and somatic mutations were called using both VarScan2 (Washington University, MO) and MuTect (Broad Institute, MA), with the intersection of the results these two methods identifying unambiguous mutations for further analysis [47, 48].

**Fig 4:**
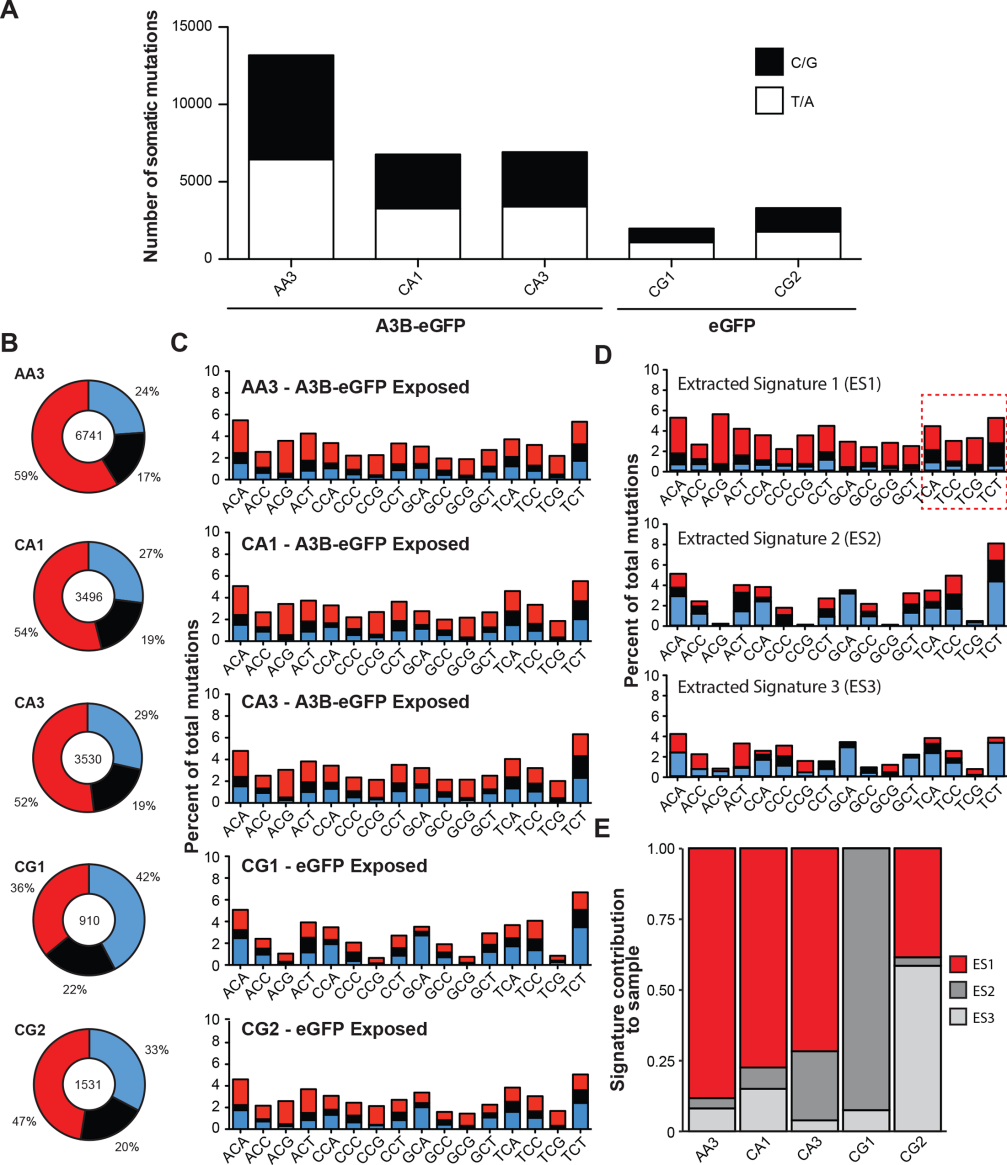
Summary of somatic mutations detected by WGS. (A) Stacked bar graphs representing total number of C/G and T/A context somatic mutations in the indicated granddaughter subclones (black and white bars, respectively). Sequences from granddaughter clone AG3 were used as a baseline to call mutations in AA3 (i.e., mutations for AG3 are not shown in bar format because WGS data from another control granddaughter clone were not available for comparison). (B) Pie charts representing the proportion of each type of cytosine mutation across the genome in the indicated granddaughter clones. Red, blue, and black wedges represent C-to-T, C-to-A, and C-to-G mutations, respectively. (C) Stacked bar graphs representing the observed percentage of C-context somatic trinucleotide mutations detected in each granddaughter clone from the B panel. (D) Stacked bar graphs representing the extracted mutation signatures from WGS data. (E) The relative proportion that each extracted mutation signature contributes to the overall base substitution spectrum in the indicated granddaughter clones.

Using this conservative approach for mutation identification, a total of 6741, 3496, and 3530 somatic mutations occurred at cytosines in granddaughter clones that had been subjected to 10 rounds of A3B-eGFP pulses in comparison to only 910 and 1531 cytosine mutations in the eGFP controls, consistent with the results of the SNP analyses described above (p = 0.018, Student’s t-test; **Fig 4A**; **S1 Table**). In particular, the A3B-eGFP pulsed granddaughter clones had higher proportions of C-to-T mutations than the eGFP controls, 59%, 54%, and 52% versus 36% and 47%, respectively (red slices in pie graphs in **Fig 4B**). The A3B-eGFP pulsed granddaughter clones also had higher proportions of mutations at A/T base pairs suggesting that genomic uracil lesions introduced by A3B may be processed by downstream error-prone repair processes analogous to those involved in AID-dependent somatic hypermutation of immunoglobulin genes [49] (**Fig 4A**).

However, despite finding significantly higher base substitution mutation loads in A3B-eGFP pulsed granddaughter clones, the overall distributions of cytosine mutations within the 16 possible trinucleotide contexts appeared visually similar for the A3B-eGFP and eGFP controls (histograms comparing the absolute frequencies of cytosine mutations with the 16 possible trinucleotide contexts are shown in **Fig 4C**). This result was initially surprising because we had expected obvious differences between the A3B-induced mutation spectrum and that attributable to other mechanisms, particularly within 5’TC contexts. However, a closer inspection of the eGFP control data sets strongly indicated that this 293-based system has a mutator phenotype possibly due to a defective replicative DNA polymerase proofreading domain and/or compromised post-replication mismatch repair [50, 51]. For instance, the eGFP controls had large numbers base substitution mutations (predominantly C-to-A, C-to-T, and T-to-C) as well as hallmark mutation asymmetries consistent with reported mutation spectra in mismatch repair defective tumors with microsatellite instabilities (**S1 Fig**) [5, 37, 51]. Moreover, each eGFP control had over 10,000 insertion/deletion mutations ranging in size from 1 to 46 base pairs (constrained by the length of the Illumina sequencing reads).

Therefore, to distinguish the A3B-eGFP induced mutation contribution from those caused by intrinsic sources, we used nsNMF decomposition via the Somatic Signatures R package to extract mutational signatures from granddaughter clones (**Methods**). This method extracted three signatures that explain 99.6% of the total variance in the observed mutation spectra. Extracted signature 1 (ES1) had large proportions of C-to-T mutations compared to the raw profiles observed for each sample. ES1 also contained low proportions of C-to-A mutations. The contribution of this signature to the overall mutation profile was specifically enriched in the A3B-eGFP pulsed granddaughter clones, contributing about 75% of all mutations (**Fig 4E**). Notably, this signature shows significant enrichments for C-to-T mutations within 5’TCG motifs, which are biochemically preferred by recombinant A3B enzyme [13, 14, 20] (Fisher’s exact test for ES1 using the average of the total observed mutations across A3B-eGFP pulsed clones: TCA, p = 0.17; TCC, p = 1.00; TCG, p < 0.0001; TCT, p = 0.017). Moreover, strong enrichments for C-to-G transversion mutations were evident for cytosine mutations within TCW contexts (W = A or T) in ES1 in comparison to other trinucleotide combinations (p = 0.0001, Student’s t-test). C-to-G transversions are hallmark A3B-mediated mutations because other known cytosine-biased mutational processes such as aging (spontaneous deamination of methyl-cytosines in 5’CG motifs) and UV-light (polymerase-mediated bypass of cross-linked pyrimidine bases) primarily result in C-to-T transitions [2, 52]. Extracted signatures 2 (ES2) and 3 (ES3) were characterized by large proportions of C-to-A mutations occurring independently of trinucleotide motif, in contrast to ES1. These WGS studies demonstrated increased genome-wide mutagenesis attributable to A3B, even over top of significant pre-existing mutation processes in this human 293 cell-based system.

## Discussion

A3B is emerging as a significant source of somatic mutation in many different cancer types (reviewed by [1–4] and see Introduction for references to primary literature). Here, we further develop a 293-based cellular system for conditional, Dox-mediated expression of A3B. The system was validated using flow cytometry, immunoblotting, enzyme activity assays, and, most importantly, three complementary mutation detection methods (3D-PCR, SNP array, and WGS). Our results demonstrated higher levels of cytosine-focused mutations in A3B-eGFP expressing cells, in comparison to eGFP controls. In particular, C-to-T transition mutations and C-to-G transversion mutations in A3B preferred trinucleotide motifs predominated after the composite mutation spectra were extracted into 3 separate signatures. These studies fortify the conclusion that A3B is a potent human genomic DNA mutagen.

An even more complex picture emerged by comparing the A3B-induced mutation signature with previously defined signatures [5]. ES1, which is attributable to A3B induction in this 293-based experimental system, clustered most closely to signature 1B, which is characterized by a dominant proportion of C-to-T transitions at NCG motifs attributed to spontaneous deamination of methyl-cytosine bases, rather than signatures 2 or 13, which are normally attributed to APOBEC. A previous study overexpressed A3B in a different 293-based system, and observed a similarly complex cytosine mutation distribution [22]. It is therefore possible that the intrinsic preference of A3B for deaminating TCA and TCG motifs may be skewed in living cells by downstream repair pathways or other mutation generating processes. In addition, although the 293-based system used here showed evidence for some sort of repair deficiency (below), ES2 and ES3 appeared most similar to signatures 5 and 16, which currently have no known etiology. Thus, the WGS data from this 293-based system indicated that the overall “APOBEC” signature is likely to be more complex than inferred by prior studies.

An unexpected outcome of our studies was the discovery of a significant preexisting mutation process operating in this 293-based system. It is likely attributable to a defect in replicative DNA polymerase proofreading function and/or in mismatch repair evident by microsatellite instability and pronounced base substitution mutation biases. However, the molecular nature of this defect is not obvious and may be genetic and/or epigenetic. For instance, the WGS data show 6 exonic and over 100 intronic alterations to mismatch repair and related genes that could induce such a mutator phenotype. These results are consistent with a prior WGS study that found 1000’s of mutation differences between 6 different 293-derived cell lines, as well as significant down-regulation of MLH1 and MLH3 in a subset of lines [53]. Our studies are also consistent with at least two additional prior reports characterizing the related 293T cell line as mismatch repair defective [54, 55]. Regardless of the precise molecular explanation, given the large number of labs worldwide that rely upon 293 or 293-derived cell lines, knowledge of this mutator phenotype is likely to be helpful for informing future experimental designs using this system.

Despite a compelling case for A3B in cancer mutagenesis (key results cited in **Introduction**), the overall APOBEC mutation signature in cancer cannot be explained by A3B alone, because it is still evident in breast cancers lacking the entirety of the *A3B* gene due to a common deletion polymorphism [56]. One or more of the other APOBEC family members with an intrinsic preference for 5’TC dinucleotide substrates may be responsible. A leading candidate is A3A due to high catalytic activity in biochemical assays, nuclear/cell-wide localization in some cell types, propensity to induce a DNA damage response and cell death upon overexpression, and the resemblance of its mutation signature in model systems to the observed APOBEC signature in many cancers [13, 19, 21, 33, 39, 42, 57–63]. *A3A* gene expression may also be derepressed as a side-affect of the *A3B* gene deletion [64]. Additional studies will be needed to unambiguously delineate the identities of the full repertoire of cancer-relevant APOBEC3 enzymes, quantify their relative contributions to mutation in each cancer type, and build upon this fundamental knowledge to improve cancer diagnostics and therapeutics.

## Supporting Information

S1 Fig T-to-C mutations in all samples exhibit a DNA replication strand bias similar to that observed in MSI cancers. (PDF)

S1 Table Somatic mutations from WGS of 293-based clones. (XLSX)
