# Supplementary material for: Mutation Processes in 293-Based Clones Overexpressing the DNA Cytosine Deaminase APOBEC3B
Source: PLoS One. 2016 May 10;11(5):e0155391. doi: 10.1371/journal.pone.0155391 (PMC4862684; doi:10.1371/journal.pone.0155391)
Supplement: S1 Fig — (PDF) [file pone.0155391.s001.pdf]

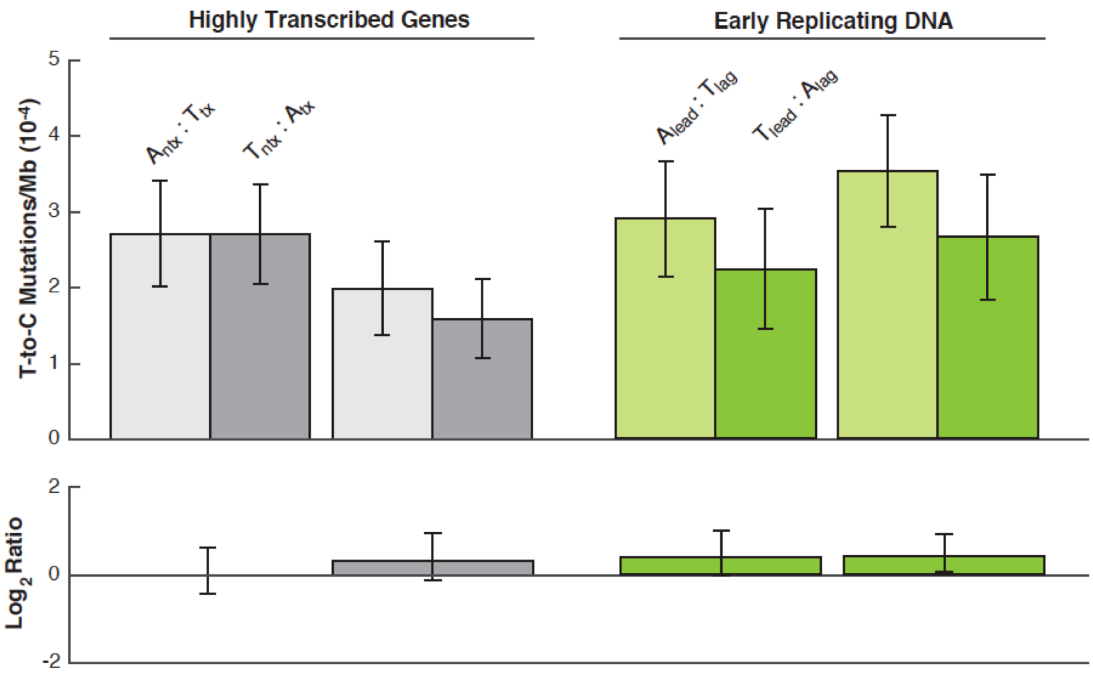

S1 Fig. T-to-C mutations in all samples exhibit a DNA replication strand bias similar to that observed in MSI cancers.

The gray bar groups represent the T-to-C mutations per megabase for genes in the highest two quartiles of transcription. The lighter bar within each group represents the mutations per megabase when "T" is on the transcribed strand (tx) and "A" is on the non-transcribed strand (ntx), and the darker bar within the group represents the inverse. Error bars represent the 95% confidence interval of these values.

The green bar groups show the T-to-C mutations per megabase that occur within the two earliest quartiles of DNA replication across the genome. The lighter bar is when "A" is on the leading strand (lead) and "T" is on the lagging strand (lag) during replication, and the darker bar represents the inverse. Error bars represent the 95% confidence interval of these values.

The lower bar graphs are the log2 ratio with 95% confidence intervals of the values in each quartile.
